# Assessment of Accuracy of Waterfall Plot Representations of Response Rates in Cancer Treatment Published in Medical Journals

**DOI:** 10.1001/jamanetworkopen.2019.3981

**Published:** 2019-05-17

**Authors:** Myung Sun Kim, Vinay Prasad

**Affiliations:** 1 Division of Internal Medicine, PeaceHealth Medical Group–Oregon, Eugene; 2 Division of Hematology and Medical Oncology, Knight Cancer Institute, Oregon Health & Science University, Portland; 3 Department of Public Health and Preventive Medicine, Oregon Health & Science University, Portland

## Abstract

**Importance:**

Response rates are a well-recognized outcome of clinical trials and provide an objective measure of drug activity.

**Objectives:**

To quantify the difference between objective response rate and visual representation of response in waterfall plots in recent articles in major medical journals and to assess the change in frequency over time with which waterfall plots are used.

**Design, Setting, and Participants:**

In a cross-sectional study, original articles of 6 top journals between July 2016 and June 2018 were manually reviewed to identify articles including a waterfall plot to describe a treatment effect of cancer therapy. Response rates visually represented in waterfall plots were compared with response rates reported as study outcomes. The number of original articles with a waterfall plot as a percentage of total original articles was evaluated, sampling articles from January, February, and March for the years 2004, 2008, 2012, 2016, and 2018.

**Main Outcomes and Measures:**

Difference between response rates depicted in waterfall plots and response rates reported as study outcomes.

**Results:**

One hundred twenty-six articles were selected for analysis. Of the 97 articles reporting investigator-assessed response rates, waterfall plots showed response rates a median (interquartile range) of 6.1% (1.8%-12.0%) higher than rates derived from investigator assessment. Forty-two articles reported response rates based on central assessment as an outcome, and waterfall plots showed response rates a median (interquartile range) of 12.0% (7.7%-18.5%) higher compared with centrally assessed response rates. The estimated percentage of original articles using waterfall plots increased from 0% in 2004 to 7% in 2018.

**Conclusions and Relevance:**

This study suggests that waterfall plots are becoming more common in oncology literature. Waterfall plots provide a visual overestimate of response rate of cancer therapies and should be evaluated with caution.

## Key Points

**Question:**

How accurate are waterfall plots in representing overall response rates reported in clinical trials?

**Findings:**

In this cross-sectional study of 126 studies published in 6 journals, waterfall plots showed visual response rates that were 6.1% higher compared with response rates based on investigator review and 12.0% higher compared with response rates based on central review. Use of waterfall plots has increased from 0% of original articles in 2004 to 7% in 2018.

**Meaning:**

This study suggests that waterfall plots are used more frequently over time and exaggerate the visual estimate of the response rate.

## Teaser

This cross-sectional study of oncology articles in high–impact factor journals examines the frequency with which waterfall plots are used and measures the accuracy of these plots in representing response rate compared with investigator-assessed and centrally assessed rates.

## Introduction

Waterfall plots are an ordered histogram depicting the best percentage change in tumor size with positive values representing increase in size of tumor and negative values representing shrinkage of tumor. Each vertical column represents a single patient. Columns are arranged from highest to lowest value from left to right resulting in a downward flowing pattern. Waterfall plots are often used to provide a concise overview of how well a group of patients respond to a novel therapy. These figures provide data on each individual patient’s best subsequent scan in a single graph.

Overall response rate is the proportion of patients who respond to a treatment based on objective criteria. The Response Evaluation Criteria in Solid Tumors (RECIST) 1.1 is a widely used set of rules to define response to treatment in solid tumors. It defines response based on the sum of the longest diameter of target lesions that are measured before initiating treatment. Best overall response is defined as the best response between start of treatment and progression of disease. Treatment response is classified as progressive disease, stable disease, partial response, and complete response based on the percentage change in the sum of the longest diameter of target lesions. More than 30% decrease is considered partial response and disappearance of target lesions is considered complete response. Objective response is reached if patients achieve partial or complete response by measurement that is confirmed by a repeated radiologic assessment no less than 4 weeks apart. In contrast, a waterfall plot displays the single best subsequent scan result for each patient able to be assessed.^1^

Waterfall plots have become a favored method of presenting results and appear often in presentations, abstracts, and published articles in oncology. Prior research has suggested that waterfall plots may be subject to interreader variation, with variability in the final plot based on the particular scorer or reader of tumor measurements.^2^ However, to our knowledge, there has not been a prior study documenting the rate of use of waterfall plots in original articles and evaluating whether their visual representation corresponds to the RECIST 1.1 response rate or other objective response criteria based on visual assessment.^2^ We set out to investigate these issues.

We sought to examine (1) the rate with which waterfall plots appeared in original articles in the top oncology journals and (2) the difference between the visual appearance of response rate in waterfall plots and the reported response rate based on investigator and/or central assessment. Central assessment is based on readings by independent radiologists as opposed to investigators who may be aware of clinical course when evaluating tumor response.

## Methods

### Data Set

We reviewed all original articles from selected medical journals in the fields of general medicine and oncology. General medical journals included in the study were *New England Journal of Medicine, JAMA,* and *Lancet*. Journals in the field of oncology were *Lancet Oncology, JAMA Oncology,* and *Journal of Clinical Oncology*. These journals were selected because they represent the top 3 general medical and oncology journals by 2017 impact factor that publish original articles in oncology. This method is similar to other investigations.^3^ Original articles included publications reporting primary data from clinical trials and also reports of post hoc analysis or pooled analysis of such data. Observational studies or systemic reviews were not included. Articles were reviewed as original articles if the content met the above criteria even if they were published as research letters or brief reports. This study of published research reports did not involve patient health data and was not submitted for institutional board review.

First, we sought to sample the literature at multiple points to assess what percentage of original articles contain waterfall plots. To do so, all original articles were selected from issues published in January, February, or March of the years 2004, 2008, 2012, 2016, and 2018 in these journals and the number of articles reporting at least 1 waterfall plot in the main text were compared with the number of all original articles.

**Figure 1.**
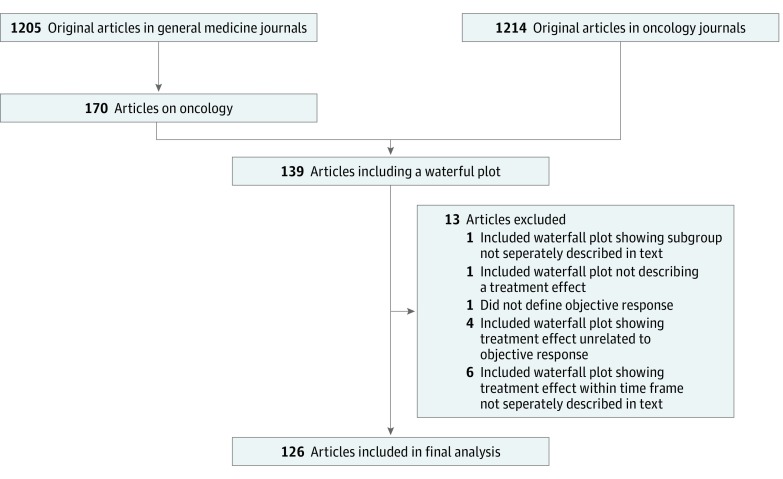
Diagram of Study Selection

Second, we sought to compare the visual estimate of response rate from waterfall plots against the (1) investigator-assessed and/or (2) centrally assessed response rate reported in the article. For this, a single investigator hand searched all original articles in these journals between July 1, 2016, and June 30, 2018, to identify all articles that report at least 1 waterfall plot in the main text. Studies were eligible if they described the efficacy of an antitumor agent either alone or in combination with other agents delivered by oral, parenteral, or intralesional route given in the primary, adjuvant, or neoadjuvant setting. Of these articles, we selected studies that (1) provided a clear definition of clinical response that included a visually estimated parameter, (2) described the number of patients showing clinical response in the results of the study, and (3) used a waterfall plot to represent individual response to treatment that correlated with clinical response. If several waterfall plots were included in a single article, each waterfall plot was assessed separately and excluded if it did not meet the above criteria. Waterfall plots were also excluded for the following reasons: (1) represented a subgroup of patients without reported response rate, (2) visualized response at point in time prior to study completion, and (3) included only patients already presented in another waterfall plot of the same article with identical response criteria in the y axis. Study selection is shown in Figure 1.

### Data Extraction

**Figure 2.**
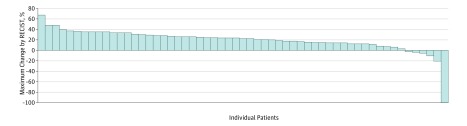
Sample Waterfall Plot From *JAMA Oncology* Adapted from Dickson et al.^4^ RECIST indicates Response Evaluation Criteria in Solid Tumors.

We then compared the response rate based on response criteria used by authors with the response rate of waterfall plots visualized as a percentage of horizontal bars of the waterfall plot falling beneath the percentage threshold for partial or complete response. To assess the response rate from waterfall plots, we counted bars. Figure 2 is adapted from an article in *JAMA Oncology* to give an example.^4^ There are 57 columns in Figure 2 and only 1 column is below the −30% threshold for response. The visualized response rate is calculated as 1 divided by 57. When only the outer contour of the histogram was shown without lines separating each column, we used computer measurements of the width of the histogram and width of the columns below the threshold to calculate the response rate. In the example of Figure 2, this would be calculated as (width of a single column)/(total width of histogram).

The objective response rate of the study was subtracted from the visualized response rate of the waterfall plot. Two separate data sets were made for investigator-assessed response and centrally assessed response. If both investigator response rate and central response rate were reported in the study, the waterfall plot was included in both data sets.

### Statistical Analysis

The median and interquartile range were calculated for both data sets using the Excel (Microsoft Corporation) function median and quartile.exc. A χ^2^ test for normality showed nonnormal distribution. Confidence intervals were not calculated because our study data included all articles of top journals within defined period and does not represent a random sample of a larger group of publications.

## Results

### Frequency of Waterfall Plots in High–Impact Factor Journals

In a sample of top journals from 2004 to 2018, we found an increase in the frequency that waterfall plots were used in original studies. None of the journals included waterfall plots in the sampled time frame in 2004. This increased to 22 of 298 original articles (7%) including a waterfall plot in 2018. In 2008, 2012, and 2016 this number was 5 of 299 (1.7%), 14 of 273 (5.1%), and 10 of 300 (3.3%). By individual journal, the articles including waterfall plots in the first 3 months of 2018 were 1 of 49 (2.0%) for *New England Journal of Medicine*, 0 of 59 (0%) for *JAMA*, 2 of 46 (4.3%) for *Lancet*, 1 of 44 (2.2%) for *JAMA Oncology*, 6 of 30 (20%) for *Lancet Oncology*, and 12 of 70 (17.1%) for *Journal of Clinical Oncology*.

### Visual Response Rate of Waterfall Plots vs Reported Response Rate

Between 2016 and 2018, we identified 126 articles that included a waterfall plot to present treatment effects of an intervention for oncologic conditions. Six articles were from general medicine journals and 120 articles were from journals in oncology. Most studies were for phase 1 or phase 2 clinical trials. The median (interquartile range) number of study participants was 60 (32-136). Of 126 trials, 100 were nonrandomized, and 89 were industry sponsored (Table).

**Table.** Characteristics of Studies From 2016 to 2019 With at Least 1 Waterfall Plot

Characteristics	Studies (N = 126)
Phase, No. (%)	
1/2	11 (9)
1	44 (35)
2	60 (48)
3	11 (8)
Randomization, No. (%)	
Yes	26 (21)
No	100 (79)
Sponsor of trial, No. (%)	
Industry	89 (71)
Academic	33 (26)
Other	4 (3)
Participants, median (IQR), No.	60 (2-136)

Abbreviation: IQR indicates interquartile range.

**Figure 3.**
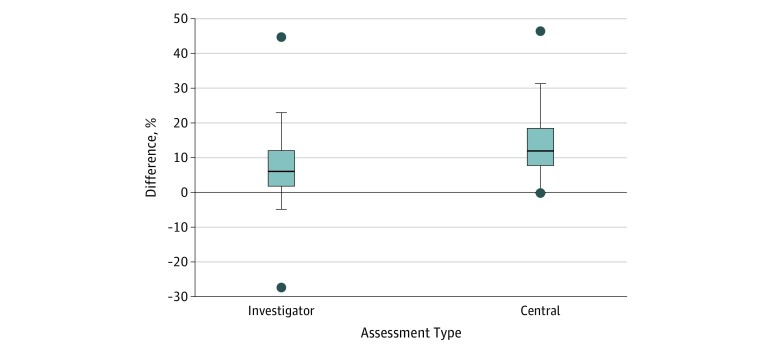
Difference Between Visual Response Rate of Waterfall Plot and Objective Response Rate From Investigator Assessment and Central Assessment Boxes indicate first to third quartile; center line, the median; whiskers, range of data points within upper (third quartile + 1.5 × interquartile range) and lower (first quartile − 1.5 × interquartile range) limits; and points, maximum and minimum data point.

Overall response rate based on investigator assessment was reported in 97 articles. Central assessment with independent review was included in 42 articles. A total of 211 waterfall plots were analyzed. Visual response rate based on waterfall plot was a median (interquartile range) of 6.1% (1.8%-12.0%) higher than the objective investigator-assessed response rate. The median (interquartile range) difference between visual response rate and centrally assessed objective response rate was 12.0% (7.7%-18.5%). Figure 3 shows differences between the visual response rate of waterfall plots and objective response rate from investigator assessment and central assessment.

## Discussion

The use of waterfall plots to visually convey the benefit seen in cancer clinical trials has gained popularity over time. Because these plots are increasingly shared on social media and used for advertisement purposes, they may provide patients and doctors with an approximation of how well a therapy is likely to work. For this reason, whether and to what extent they exaggerate true response rate is worth noting. In our study, we found that waterfall plots exaggerate response rate 6.1% over investigator-assessed rates and 12.0% over centrally assessed rates.

There are likely 2 reasons for our findings. First, RECIST 1.1 requires a confirmatory scan documenting more than 30% reduction in tumor measurements to count as a response, while waterfall plots show the single best subsequent scan.^5,6^ For this reason, not every bar below the −30% line is a response, and some plots color-code patients as having stable disease, partial response, and so on. Second, a response rate is calculated based on a true intention-to-treat denominator, while the waterfall plot only includes patients able to be assessed for response. Patients lacking postbaseline scans are not evaluable for response and, thus, are excluded from the waterfall plots. Causes of dropout when specified range from discontinuation of therapy (death, clinical deterioration, toxic effects), withdrawal of consent, missing postbaseline scans, and so on. Thus, some patients with rapid progression or death may be excluded.

There are several potential remedies for the visual exaggeration of waterfall plots. First, investigators may provide a plot of the second-best scan for all study participants. Second, investigators could include columns for patients unable to be assessed. Third, investigators could provide waterfall plots at landmark times, eg, 12-week waterfall plot, 24-week waterfall plot, and so on. This would provide the tumor assessment for all patients at this milestone. Here, too, patients unable to be assessed can be added.

Our review of recent original articles shows that there is a difference between the overall response rate reported in a study and the visually represented response rate in waterfall plots. This difference is more pronounced when response rate is based on independent review, which is considered more objective than investigator review. Considering that response rate is considered a key outcome in reporting efficacy of novel therapies, it is concerning that waterfall plots represent an overstatement of results in many cases. With an increasingly large number of therapies competing for attention and resources, the perception of clinically significant antitumor activity is often critical in securing future funding and approval.

Prior work^2^ has focused on rates of interreader variation in the construction of waterfall plots, and our article adds to these concerns, noting that reliance on a single best subsequent measurement biases a waterfall plot toward a more favorable estimate of a therapy’s activity. Coupling these 2 findings suggests that a reporting system that uses a measurement with variability and always selects the single best subsequent result will tend to upwardly bias an estimate. This conclusion likely has implications in biomedicine that extend beyond oncology.^7^

### Limitations and Strengths

There are several limitations to our study. First, we focused on contemporary clinical trials published in high–impact factor medical journals, but waterfall plots are used in many journals, conferences, and trade publications; thus, the relationships we identify may be different in other sources. Second, we counted columns by hand if at all possible, but in a number of instances, when columns were not discrete, we relied on computer measurements. Ironically, this raises concern that our study may suffer from issues of variability in measurement, just as scans of solid tumors do. However, this was just a fraction of included studies, and omission of these articles would not materially change our conclusions.

There are several strengths to our study. Our study gives a broad overview of the recent patterns of use of waterfall plots in oncology. It also quantifies the degree to which the visualized response rate deviates from reported overall response rates. The findings are meaningful in the context of increasingly frequent use of waterfall plots as shown in this study.

## Conclusions

We found that waterfall plots occur more frequently in the biomedical literature over time and that they visually bias the estimate of response rate upward. Given the widespread use of these figures in framing the discussion around cancer therapy, our findings provide an important, cautionary note. To maintain the utility of waterfall plots while preserving the integrity of reporting outcomes of clinical trials, we suggest clear statements about dropout rates and reasons for dropout. Waterfall plots may evolve to include missing data points to avoid misrepresentation of response rates. In addition, we suggest use of landmark plots and second-best scans, as well as a clear statement about the RECIST 1.1 response rate.
